# Autonomy Need Dissatisfaction in Daily Life and Problematic Mobile Phone Use: The Mediating Roles of Boredom Proneness and Mobile Phone Gaming

**DOI:** 10.3390/ijerph17155305

**Published:** 2020-07-23

**Authors:** Wei Hong, Ru-De Liu, Yi Ding, Rui Zhen, Ronghuan Jiang, Xinchen Fu

**Affiliations:** 1 Beijing Key Laboratory of Applied Experimental Psychology, National Demonstration Center for Experimental Psychology Education (Beijing Normal University), Faculty of Psychology, Beijing Normal University, Beijing 100875, China; psyhongwei@163.com (W.H.); jrh_psy@163.com (R.J.); fxc_psy@163.com (X.F.); 2 Graduate School of Education, Fordham University, New York, NY 10023, USA; yding4@fordham.edu; 3 Institute of Psychological Sciences, Hangzhou Normal University, Hangzhou 311121, China; zhenrui1206@126.com

**Keywords:** autonomy need dissatisfaction, problematic mobile phone use, boredom proneness, mobile phone gaming, multiple mediation

## Abstract

Psychological needs dissatisfaction has been identified as hindering adaptive development, in which autonomy need dissatisfaction, as one core component, may be associated with adolescents’ maladaptive online behaviors. Sporadic research has examined the association between autonomy need dissatisfaction and problematic mobile phone use (PMPU). Boredom proneness and mobile phone gaming were suggested to be linked to this association. This study aimed to examine the mediating effects of boredom proneness and mobile phone gaming in the association between autonomy need dissatisfaction and PMPU. A total of 358 secondary school students completed questionnaires at three waves; autonomy need dissatisfaction was measured in time 1 (T1); boredom proneness and mobile phone gaming were measured one year later (time 2, T2); PMPU was measured two years later (time 3, T3). The structural equation model results showed that T1 autonomy need dissatisfaction not only directly predicted T3 PMPU, but also exerted effects via the mediating role of T2 boredom proneness and the chain mediating role of T2 boredom proneness and T2 mobile phone gaming. These findings reveal the unique role of specific psychological need in engaging PMPU, which provides support to targeted interventions, such that promoting autonomy need satisfaction may be an instrumental procedure to prevent adolescents from addiction-like online behaviors.

## 1. Introduction

Mobile phones, as the most accessible device to connect to the internet, have penetrated every aspect of daily lives, such that they help people obtain information, maintain social connectedness, and entertain themselves [1,2]. According to a national survey in China, there were 897 million mobile phone users as of March 2020, accounting for 99.3% of the Internet users [3]. Some of the users invested an excessive amount of time and resources into their mobile phones. This behavior can be described as problematic mobile phone use (PMPU), which refers to a constellation of emerging addiction symptoms, including cravings, withdrawal, and loss of control [4]. Numerous negative consequences occur after engaging in PMPU. For instance, PMPU has been identified to lead to sleep problems [5,6], poor academic performance and school adjustment [7,8], cognitive failures [6,9], and physical and mental health problems [10,11,12]. Furthermore, it was found that 10% of British adolescents were problematic users and 20.5% of them were potential problematic users [13]. A recent research showed that the prevalence of PMPU was 29% in young adults from the United Arab Emirates [1]. Such potential hazards and the high prevalence of PMPU stimulate public concerns and gain increasing scholarly attention.

People are active and purposive when engaging on the internet via mobile phones because it can satisfy specific psychological needs, as postulated by uses and gratification theory [14]. This perspective implies that people with unsatisfied needs in daily life tend to use mobile phones as a compensator to cope with the negative life situation. Combined with the model of compensatory internet use [15], this compensatory use of the internet via mobile phones is more likely to result in problematic use and addiction tendencies. A substantial body of literature has revealed that psychological needs dissatisfaction in daily life contributes to pathological Internet use (PIU) [16] and PMPU [17].

Based on self-determination theory, humans have three inherent psychological needs, including the need for autonomy, competence, and relatedness. Deci and Ryan [18] proposed that psychological needs satisfaction is an essential nutriment for psychological growth and wellness; its dissatisfaction hinders self-integrity and lead to problematic outcomes. Given that different needs play different roles in behavior patterns and social functioning [19], an increasing number of studies attempted to differentiate the unique effect of each type of need. One of the very few studies found that only autonomy (not relatedness and competence) need dissatisfaction significantly predicted problematic online behaviors [20]. It is known that autonomy need is described as the need to self-regulate their experiences and actions [21]. That is, when behaviors are volitional and self-endorsed, individuals would experience high levels of autonomy need satisfaction. Stated differently, autonomy need dissatisfaction suggests what individuals do is not congruent with their intrinsic motivation and authentic interests. As a result, ameliorating behaviors, such as engaging on the internet through mobile phones, are activated to compensate for the lack of fulfillment of this kind of need, which increases the probability of problematic use [15]. In short, it seems that autonomy need dissatisfaction is positively associated with PMPU.

Furthermore, autonomy need dissatisfaction as the perceptions of the external environment can be considered to be a distal factor in explaining the etiology of addictive symptoms of PIU; these distal factors exert effects on maladaptive online behaviors via the mediating effects of proximal factors, as postulated in the cognitive-behavioral model of PIU [22]. For instance, boredom proneness results from the external environment without autonomy [23], and servers as a contributor to PMPU [24,25]. Similarly, online gaming, as a specific behavioral response to cope with autonomy need dissatisfaction, is an important predictor of PMPU [26]. These relations indicate that boredom proneness and mobile phone gaming may be potential mediators in the process. However, there has been a lack of empirical research to support this relation. To address this issue, this study aimed to examine the mediating roles of boredom proneness and mobile phone gaming in the association between autonomy need dissatisfaction in daily life and PMPU.

### 1.1. Boredom Proneness as a Mediator

Boredom proneness may be a potential mediator between autonomy need dissatisfaction and PMPU. Specifically, boredom refers to a general tendency to experience boredom in situations with deficits in interest, meaning, excitement, and challenge [27]. People with autonomy need dissatisfaction have relatively few opportunities to make their own decisions, and have to engage in activities incongruent with their authentic interests [21]. Thus, non-interest-orientated activities may lead to low levels of psychological arousal and high levels of boredom proneness [28]. This notion has been supported by the various findings that psychological needs (including autonomy need) satisfaction/dissatisfaction significantly predicts boredom in sports activities [29], in academic settings [30], and in work domains [31]. In a 2009 study, adolescent soccer athletes who perceived less autonomy reported more boredom experience [23]. Thus, autonomy need dissatisfaction in daily life seems to be positively associated with boredom proneness.

Regarding the second stage of the mediation process, boredom proneness has been identified as a high-risk factor for PIU [32,33] and PMPU [24,34]. Adolescents with high levels of boredom proneness tend to experience low levels of internal motivation and external stimulation [27]. One approach to cope with boredom is to engage in online activities as they may help to increase the feelings of excitement and sensation [32,35]. Chronic and habitual use of this approach would increase the risk of engaging in PIU [32,33]. Similarly, previous research has found that boredom proneness positively predicts PMPU among adolescents [24,34]. Altogether, it appears that autonomy need dissatisfaction is positively associated with boredom proneness, which in turn is positively associated with PMPU.

### 1.2. Mobile Phone Gaming as a Mediator

Another potential mediator may be mobile phone gaming, because distal causes and proximal factors jointly facilitate an excessive use of specific internet functions (e.g., online gaming), which further leads to behavioral symptoms of PIU [22]. As stated earlier, individuals with autonomy need dissatisfaction may activate ameliorating behaviors to compensate for the deficits in this kind of need. As reviewed by Ryan and Deci [21], a key characteristic of games is providing opportunities for actions. For instance, players are free to choose the types of games and activities that they want to engage in, to decide the avatars and roles, and to fulfill the game missions. Experimental evidence has indicated that the autonomy character of a game would facilitate immersion-related experiences, further increasing enjoyment and decreasing boredom in the game world [36]. In this sense, people who experience autonomy need dissatisfaction might have the motivation to engage in gaming as a way to compensate, thus exhibiting longer game-playtime on a weekly basis [37]. More important, empirical research has found that psychological needs dissatisfaction [38] and autonomy need dissatisfaction [20] in the real world positively predict the excessive use of video games. Thus, autonomy need dissatisfaction appears to be positively associated with frequent mobile phone gaming.

Moreover, when adolescents have a history of mobile phone use for gaming, desirable game experiences (e.g., flow experience in the game world) may encourage them to repeatedly engage in this activity [39]. In the long run, they are more likely to frequently act on mobile phones and become addicted to using mobile phones [40]. In support of this notion, frequent online gaming has been shown to positively predict PIU in cross-sectional research [41] and predict PIU one year later in the longitudinal research [42]. Similarly, Jeong, Kim, Yum and Hwang [26] and Lee, Kim and Choi [40] found that frequent mobile gaming contributed to PMPU. Altogether, it appears that autonomy need dissatisfaction is positively associated with mobile phone gaming, which in turn is positively associated with PMPU.

### 1.3. A Multiple Mediation Model

The mediating roles of boredom proneness and mobile phone gaming have been advanced to describe the relation between autonomy need dissatisfaction and PMPU wherein boredom proneness was argued to positively associate with frequent mobile phone use [43]. For instance, Chou, et al. [44] found that adolescents with high boredom proneness are more easily to perceive low levels of external stimulation and are more likely to engage in online gaming for self-entertainment. Likewise, Biolcati, Mancini and Trombini [25] supported this finding and found that adolescents with higher boredom proneness reported higher levels of participation in mobile phone gaming in comparison to adolescents with lower boredom proneness.

Taken together, individuals with autonomy need dissatisfaction in the real world cannot voluntarily make choices and engage in activities congruent with their authentic interests [18]. Thus, they are prone to having low intrinsic motivation and exhibit low psychological involvement, which may increase the tendency to experience boredom [21,45,46]. Furthermore, bored individuals are more likely to play mobile games as a compensator of boredom [15]. In this regard, frequent mobile phone gaming increases the risk of problematic use and addictive symptoms [26,40]. Accordingly, it is possible that autonomy need dissatisfaction is indirectly associated with PMPU via the multiple mediating role of boredom proneness and mobile phone gaming.

### 1.4. The Present Study

**Figure 1:**
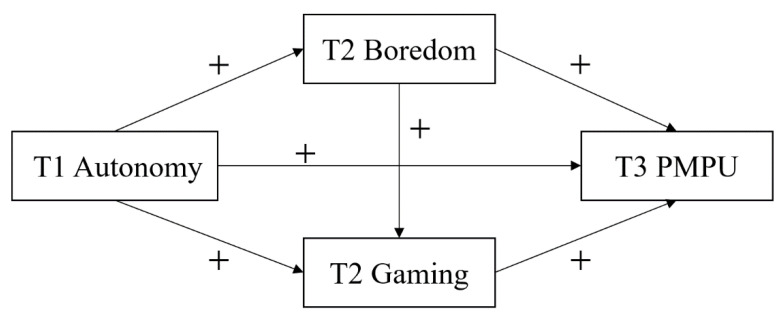
The conceptual model between autonomy need dissatisfaction and PMPU. Note. T1 Autonomy = Autonomy need dissatisfaction in Time 1; T2 Boredom = Boredom proneness in Time 2; T2 Gaming = Mobile phone gaming in Time 2; T3 PMPU = Problematic mobile phone use in Time 3.

According to the above findings, autonomy need dissatisfaction in daily life has been argued to be a contributing factor to PMPU. Autonomy need dissatisfaction as the perceptions of the external environment may exert effects on psychological symptoms via individual characteristics. Based on self-determination theory [18], the model of compensatory internet use [15], and the cognitive-behavioral model of PIU [22], boredom proneness as a dispositional factor can be partially attributed to the lack of autonomy from the external environment; and gaming involved a specific mobile phone usage can be considered as coping strategies. That is, boredom proneness and mobile phone gaming can be postulated as potential mediators to elucidate how autonomy need dissatisfaction was associated with PMPU. Nevertheless, there has been a lack of empirical research, especially using cross-temporal designs, to examine whether autonomy need dissatisfaction is associated with PMPU via the mediating roles of boredom proneness and mobile phone gaming. To this end, we attempted to assess the independent variable in Time 1 (T1), the mediating variables in Time 2 (T2), and the dependent variable in Time 3 (T3). As shown in Figure 1, this study was guided by the following hypotheses:

**H1:**  *T1 autonomy need dissatisfaction in daily life is positively associated with T3 PMPU.*

**H2:**  *T2 boredom proneness mediates the association between T1 autonomy need dissatisfaction and T3 PMPU.*

**H3:**  *T2 mobile phone gaming mediates the association between T1 autonomy need dissatisfaction and T3 PMPU.*

**H4:**  *T1 autonomy need dissatisfaction in daily life is indirectly associated with T3 PMPU through the multiple mediating role of T2 boredom proneness and T2 mobile phone gaming.*

## 2. Materials and Methods

### 2.1. Participants and Procedures

A sample of 1060 students from a regular secondary school in Beijing, China, was recruited to participate in the first data collection (T1). Due to graduation, 819 students participated in the second data collection (T2) after one year and 358 students participated in the third data collection (T3) after two years. This study focused on the participants who completed the questionnaires at three waves. The sample comprised 154 (43.0%) boys and 204 girls (57.0%). They had an average age of 13.19 years (standard deviation (*SD)* = 1.44), ranging from 12 to 16 years. Each participant reported having a constant Internet-accessible mobile phone.

This research was approved by the Academic Ethics Committee of the Faculty of Psychology at Beijing Normal University. Before the formal investigation, participants and their parents or legal guardians were provided with written consent forms, which informed them that personal information would be kept confidential and their responses would be used only for research purposes. Additionally, students were informed that they had the right to opt out of the research at any time. The research assistants distributed and collected the self-report questionnaires in the regular classrooms. Data collection took approximately 15 min, and participants were compensated with a small gift (e.g., a pen).

### 2.2. Measure

#### 2.2.1. Autonomy Need Dissatisfaction

The level of autonomy need dissatisfaction was measured in T1 by the Basic Need Satisfaction in General Scale, which consists of the domains of autonomy, competence, and relatedness needs satisfaction [47]. This scale has been tested and used in the Chinese context [48]. The autonomy subscale contains three negative items (e.g., There is not much opportunity for me to decide for myself how to do things in my daily life), which has been used to assess autonomy need dissatisfaction [49]. Participants rated the items on a 5-point Likert scale (1 = *not at all true*, 5 = *very true*), with higher scores indicating higher levels of autonomy need dissatisfaction. The internal consistency of this scale showed acceptable reliability (Cronbach α = 0.60).

#### 2.2.2. Boredom Proneness

The level of boredom proneness was measured in T2 by the short version of the Boredom Proneness Scale [27]. This scale contains eight items (e.g., I often find myself at “loose ends,” not knowing what to do) with one-dimensional structure. Participants rated the items on a 7-point Likert scale (1 = *strongly disagree*, 7 = *strongly agree*), with higher scores indicating higher levels of boredom proneness. The internal consistency of this scale showed satisfactory reliability (Cronbach α = 0.94).

#### 2.2.3. Mobile Phone Gaming

The measure of mobile phone gaming in T2 was adapted from the Chinese Internet Usage Questionnaire [50] and the Mobile Phone Use Patterns Questionnaire [43]. In total, there were 10 items regarding Internet use and 17 items regarding mobile phone use. After instructing “according to your daily routine, …”, only one item (i.e., I play mobile games on my phone) was used to assess the mobile phone gaming frequency on a daily basis. Participants rated the items on a 5-point Likert scale (1 = *never*, 5 = *always*), with higher scores indicating more frequent use for mobile games.

#### 2.2.4. Problematic Mobile Phone Use (PMPU)

Participants’ severity of PMPU was assessed in T3 by the short version of the Mobile Phone Problem Use Scale [51], which has been validated in the Chinese context and showed good validity and reliability [17,52]. This scale contains 10 items (e.g., I find it difficult to switch off my mobile phone) with five aspects, including craving, withdrawal, peer dependence, loss of control, and negative life consequences. Participants rated the items on a 5-point Likert scale (1 = *strongly disagree*, 5 = *strongly agree*), with higher scores indicating more severe PMPU. The internal consistency of this scale showed satisfactory reliability (Cronbach’s *α* = 0.87).

### 2.3. Data Analyses

Means, standard deviations, and Pearson correlations were calculated using SPSS 19.0. The hypothesized multiple mediation model was tested by structural equation modeling (SEM) using Mplus 7.1 [53]. The model was evaluated by following model fit indices: the chi-square values (χ2), the comparative fit index (CFI), the Tucker–Lewis fit index (TLI), the root mean square error of approximation (RMSEA), and the standardized root mean square residual (SRMR). The CFI and TLI at 0.90 or above, and the RMSEA and SRMR at 0.08 or lower, indicate that the model is acceptable [54].

## 3. Results

### 3.1. Descriptive Statistics and Correlations

Means, standard deviations, and Pearson correlations are presented in Table 1. As shown, autonomy need dissatisfaction was significantly and positively correlated with boredom proneness and PMPU, but it was not correlated with mobile phone gaming. Furthermore, each two elements of boredom proneness, mobile phone gaming, and PMPU had a positive association.

### 3.2. Examinations of the Measurement Model

Before testing the hypothesized model by SEM, it was necessary to examine the measurement model. According to the recommendation from Wu and Wen [55], autonomy need dissatisfaction could be loaded by the three observed items; boredom proneness that has eight items with one-dimensional structure could be parceled into three indicators; mobile phone gaming with only one item could be loaded by the one item; PMPU with five aspects could be loaded by the five substructures. Altogether, the CFA results of the measurement model showed a good model fit: χ^2^/df = 3.17, CFI = 0.95, TLI = 0.93, RMSEA = 0.08, SRMR = 0.05, in that all the loadings on latent variables were significant (*p* < 0.001).

### 3.3. Examinations of the Structural Model

**Figure 2:**
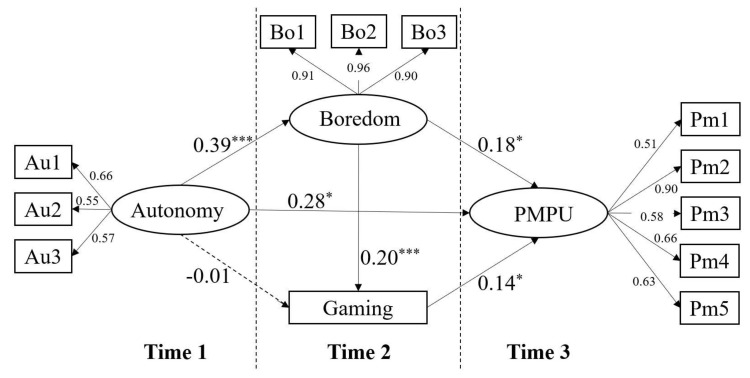
The mediation model of the association between T1 autonomy need dissatisfaction and T3 PMPU. Note. All the loadings on latent variables were significant (*p* < 0.001). Autonomy = Autonomy need dissatisfaction, Boredom = Boredom proneness, Gaming = Mobile phone gaming, PMPU = Problematic mobile phone use. * *p* < 0.05, *** *p* < 0.001.

As hypothesized, a multiple model with T1 autonomy need dissatisfaction as the independent variable, T2 boredom proneness and mobile phone gaming as the mediators, and T3 PMPU as the dependent variable was established. The SEM results showed a good model fit: χ^2^/df = 3.15, CFI = 0.94, TLI = 0.92, RMSEA = 0.05, SRMR = 0.05. As shown in Figure 2, T1 autonomy need dissatisfaction significantly predicted T3 PMPU. Similarly, T1 autonomy need dissatisfaction positively predicted T2 boredom proneness, which in turn positively predicted T3 PMPU. However, T1 autonomy need dissatisfaction did not predict T2 mobile phone gaming, although T2 mobile phone gaming positively predicted T3 PMPU.

To further examine the significance of the indirect effects, bias-corrected bootstrap tests derived with 1000 samples were used. That the 95% confidence interval did not contain zero indicated statistical significance [56]. As shown in Table 2, T1 autonomy need dissatisfaction positively predicted T3 PMPU, supporting H1. Furthermore, T2 boredom proneness significantly mediated the association between T1 autonomy need dissatisfaction and T3 PMPU, supporting H2. Whereas, mobile phone gaming did not mediate the association between T1 autonomy need dissatisfaction and T3 PMPU, rejecting H3. Additionally, the chain of T2 boredom proneness and T2 mobile phone gaming significantly mediated the association between T1 autonomy need dissatisfaction and T3 PMPU, supporting H4.

## 4. Discussion

This study focused on autonomy need dissatisfaction and examined its potential effect on PMPU. Boredom proneness and mobile phone gaming were suggested to be incorporated into this association to elucidate the underlying mechanism. Based on three-wave data, the SEM model results showed that T1 autonomy need dissatisfaction not only directly predicted T3 PMPU, but also exerted effects on T3 PMPU via the mediating role of T2 boredom proneness and via the chain mediating role of T2 boredom proneness and T2 mobile phone gaming. Altogether, the findings provide empirical evidence to support the relation between specific psychological need and PMPU, which lends further insight into targeted prevention and interventions of problematic online behaviors.

### 4.1. Autonomy Need Dissatisfaction, Boredom Proneness, and PMPU

This study demonstrated that T1 autonomy need dissatisfaction directly predicted T3 PMPU, it also indirectly predicted T3 PMPU through the mediating role of T2 boredom proneness. According to self-determination theory [18], adolescents with autonomy need dissatisfaction have few opportunities to volitionally make choices and self-organize actions in daily life. Thus, they may have to participate in activities with little intrinsic motivation, which increases a tendency to experience boredom [29,45]. For instance, they may execute what others compel them to do, such as participating in extracurricular courses that are arranged by their parents. In this sense, they are more likely to experience boredom. This finding was consistent with the previous studies that the higher levels of autonomy need dissatisfaction that adolescents perceive, the more likely they would experience boredom [31].

Furthermore, bored adolescents are more likely to seek external stimulation to cope with boredom [27], and thus they may spend much time and resources on the internet (or via mobile phones), which further increases the risk of problematic behaviors, including PIU [32,33] and PMPU [24]. Altogether, adolescents with autonomy need dissatisfaction cannot freely make decisions and volitionally engage in what they are interested in, which chronically contributes to boredom proneness. These bored adolescents are more likely to frequently act on mobile phones, leading to problematic use. Thus, it seems that autonomy need dissatisfaction in daily life gives rise to boredom proneness, which in turn increases the risk of subsequent PMPU.

### 4.2. Autonomy Need Dissatisfaction, Mobile Phone Gaming, and PMPU

This study showed that T1 autonomy need dissatisfaction did not predict T2 mobile phone gaming although T2 gaming positively predicted T3 PMPU. This finding weakly supported the mediating role of mobile phone gaming in the mediation process because the first stage was not significant. One possibility may be that psychological need dissatisfaction plays a double-edged role in determining online gaming [37]. As mentioned earlier, games that provide adolescents with opportunities for actions can assist in compensating for unsatisfied autonomy need in the real world [21,57]. Therefore, adolescents with autonomy need dissatisfaction may resort to the internet (or via mobile phones) to compensate for this dissatisfaction [15]. For instance, when individuals feel psychologically pressured and constrained, they would use mobile phones for gaming to alleviate these undesirable feelings as a way to compensate because they are free to do whatever they want in the game world. This perspective implies that the higher levels of autonomy need dissatisfaction that adolescents perceive, the more frequent mobile phone gaming they would engage in [38].

Nevertheless, adolescents with autonomy need dissatisfaction have few opportunities to decide for themselves even though they may think that gaming is tempting [39,47]. Specifically, adolescents with autonomy need dissatisfaction may be under the restrictions of their parents, particularly when engaging in mobile phone gaming. This perspective suggests that the higher levels of autonomy need dissatisfaction that adolescents perceive, the fewer opportunities they might have to play mobile games. Taken these two perspectives together, the former compensatory effect (i.e., autonomy need dissatisfaction motivates mobile gaming as a compensator) may neutralize the later restriction effect (i.e., autonomy need dissatisfaction indicates few opportunities for mobile gaming). Thus, it is not surprising that autonomy need dissatisfaction in daily life was weakly associated with mobile phone gaming. Future studies are warranted to further examine the complicated association between autonomy need dissatisfaction and online gaming.

### 4.3. A Multiple Mediation Model

One intriguing finding was that the chain of T2 boredom proneness and T2 mobile phone gaming significantly mediated T1 autonomy need dissatisfaction and T3 PMPU. Consistent with the etiology of addictive symptoms of PIU [22], psychological needs dissatisfaction as a distal factor exerts effects on addiction tendencies through the mediating variables (i.e., boredom proneness and mobile phone gaming). Specifically, adolescents with autonomy need dissatisfaction in real life tend to have relatively fewer opportunities to make decisions; thus, they may have to engage what is not congruent with their authentic interests [47]. For instance, the parents of Chinese students may arrange for them to engage in repetitive and monotonous academic activities. In a long run, they may possess low levels of intrinsic motivation and exhibit high levels of boredom proneness. Concurring with the earlier findings [25,44], bored adolescents may engage in online gaming to alleviate boredom as they can obtain external stimulation and gain flow experiences when fully involving in gaming [39]. For instance, participants reported that they played a kind of multiplayer online battle arena game named *Arena of Valor* on mobile phones because they felt that doing so can swipe away boring time. Additionally, mobile phone gaming has been identified as a high-risk factor for PMPU [26,40]. That is, adolescents who frequently engage in mobile phone gaming are at risk in developing problematic use and nurturing addiction tendencies. Taken together, it seems that autonomy need dissatisfaction in daily life positively predicts boredom proneness that contributes to frequent mobile phone gaming, which in turn leads to subsequent PMPU.

### 4.4. Limitations, Future Directions, and Implications

There are several limitations of this study. First, self-reported data may produce response bias although there was no serious common method bias using Harman’s single factor test [58]. Future studies could record time of generalized use and gaming on mobile phones, which may provide objective data and enhance reliability and validity. Second, the reliability of the measure of autonomy need dissatisfaction appeared to be somewhat low although it has been used in several studies [47,48,49]. Thus, this scale should be further improved in future research. Third, this study recruited secondary school students only from a regular secondary school; therefore, generalization of the conclusions to other groups should be made with caution. Future studies could focus on adults and/or clinical groups, which may contribute to a broader application of these findings. Fourth, the mediating effects seemed to be relatively small, however small effects can assist in developing theories when the findings support the theoretical hypotheses [59]. In addition, small effects should not be disregarded because they might be accumulated to generate large effects with the changing conditions [60]. In this digital age in particular, the use of mobile phones has exponentially grown and corresponding problems (e.g., addiction tendencies) have been increasing and appear severe, thus possibly leading to large effects on PMPU in future studies.

Despite the limitations, notable implications are twofold. From a theoretical perspective, this study was the first of its kind to use a cross-temporal design and to exclusively examine the effect of autonomy need dissatisfaction on subsequent PMPU. On the one hand, this study focused on the role of specific need (i.e., autonomy need), instead of psychological needs as a single entity, in explaining maladaptive online behaviors. On the other hand, boredom proneness as an individual characteristic and gaming as possible coping strategies helped to elucidate the potential etiology of addiction-like symptoms associated with mobile phone use in the framework of self-determination. These findings based on the three-wave data revealed that autonomy need dissatisfaction not only directly predicted subsequent PMPU, but also exerted indirect effects via the mediating roles of boredom proneness and mobile phone gaming. These findings help to develop a better understanding of the formation process of PMPU, which provides support for prevention and intervention programs. For instance, excessive parental restrictions on children’s online behaviors (e.g., limiting use time, monitoring online content) may backfire because these children perceive autonomy need dissatisfaction and may increase addiction-like tendencies [61,62]. In contrast, we recommend that families and schools provide adolescents with a certain degree of autonomy and encourage adolescents to self-organize their behaviors, which can reduce their tendencies to experience boredom. Accordingly, these less-bored adolescents are less likely to engage in high-frequency game play, thus decreasing the risk of engaging in PMPU. Additionally, families and school personnel could try to purposefully increase diverse activities to avoid boredom from adolescents, as well as to guide adolescents to increase appropriate mobile phone use and decrease excessive mobile gaming, which may be instrumental to prevent from addiction-like online behaviors.

## 5. Conclusions

This is one of very few studies to focus on the association between autonomy need dissatisfaction in daily life and PMPU. With boredom proneness and mobile phone gaming introduced, the mediation model may contribute to explaining the potential mechanism of this association. Based on three-wave data, the results showed that T1 autonomy need dissatisfaction not only directly predicted T3 PMPU, but also exerted effects on T3 PMPU via the mediating role of T2 boredom proneness and via the chain mediating role of T2 boredom proneness and T2 mobile phone gaming. Altogether, these findings reveal the unique role of specific psychological need satisfaction in PMPU, which suggests that promoting autonomy need satisfaction may prevent adolescents from mobile phone addiction.

## Figures and Tables

**Table 1:** Means, standard deviations, and correlations among the main variables.

Variables	M	SD	1	2	3	4
1 T1 Autonomy	2.91	0.84	-			
2 T2 Boredom	3.82	1.33	0.32 ***	-		
3 T2 Gaming	3.24	1.17	0.06	0.19 ***	-	
4 T3 PMPU	2.70	0.76	0.25 ***	0.27 ***	0.21 ***	-

Note. T1 Autonomy = Autonomy need dissatisfaction in Time 1; T2 Boredom = Boredom proneness in Time 2; T2 Gaming = Mobile phone gaming in Time 2; T3 PMPU = Problematic mobile phone use in Time 3; *** *p* < 0.001.

**Table 2:** Bias-corrected bootstrap tests on the direct and indirect effects.

Paths	Standardized(β)	95% Confidence Interval		Hypotheses
		Low	High	
T1 Autonomy → T3 PMPU	0.277	0.112	0.443	Supporting H1
T1 Autonomy → T2 Boredom → T3 PMPU	0.069	0.007	0.013	Supporting H2
T1 Autonomy → T2 Gaming → T3 PMPU	−0.001	−0.024	0.022	Rejecting H3
T1 Autonomy → T2 Boredom → T2 Gaming → T3 PMPU	0.011	0.001	0.025	Supporting H4

Note. T1 Autonomy = Autonomy need dissatisfaction in Time 1; T3 PMPU = Problematic mobile phone use in Time 3; T2 Boredom = Boredom proneness in Time 2; T2 Gaming = Mobile phone gaming in Time 2.
